# Progressive White Matter Injury Dominates in Patients Receiving Hemodialysis over Peritoneal Dialysis after 1 Year of Dialysis

**DOI:** 10.34067/KID.0000001158

**Published:** 2026-03-19

**Authors:** Jessica A. Vanderlinden, Arsh Jain, Christopher McIntyre

**Affiliations:** 1 Department of Medical Biophysics, Western University, London, Ontario, Canada; 2 Schulich School of Medicine and Dentistry, Western University, London, Ontario, Canada; 3 Lawson Health Research Institute, London, Ontario, Canada; 4 Lilibeth Caberto Kidney Clinical Research Unit, London Health Sciences Centre, London, Ontario, Canada

**Keywords:** clinical nephrology, cognition, dialysis, hemodialysis, imaging, longitudinal data analysis, peritoneal dialysis

Hemodialysis is known to be associated with circulatory stress, cognitive impairment, and white matter injury (WMI).^1^ Specifically, the degree of WMI is proportional to the degree of cognitive impairment, with hemodialysis cognitive impairment being associated with hemodynamic functional measures and widespread WMI damage.^2^ WMI is more comprehensively studied in hemodialysis populations than those receiving peritoneal dialysis (PD). However, there are data suggesting that PD patients exhibit imaging abnormalities in cerebral tracts that correspond with cognitive outcomes.^3^ The objective of this study was to determine the degree of WMI change after a year of dialysis in a cohort of patients receiving dialysis (hemodialysis or PD) and its association with cognitive impairment.

Patients were scanned two times (baseline and 1 year later). WMI was determined by diffusion tensor imaging (DTI) acquired using a 16-channel (12 head, four neck) magnetic resonance imaging scanner. Raw DTI images were optimized and corrected for head movement and intensity inhomogeneities. Corrected images received tensor fitting using nonlinear least squares, which resulted in fractional anisotropy (FA) scalar maps. FA maps were normalized to a Montreal Neurological Institute 152 brains Standard 1 mm T_1_-weighted template, with Tract-Based Spatial Statistics analysis being conducted to determine if there were differences between the two time points. Significant FA differences between time points were obtained *via* higher level/nontime series design for general linear models for paired groups. Further analysis gathered 20 white matter (WM) area mean skeletonized FA values using the Johns Hopkins University WM atlas. Specifically, lower FA values at 1-year time point indicates that there is reduced WM structural integrity, which corresponds to WMI. Supplemental Figure 1 shows a detailed imaging analysis description.

This single-center repeated measure observational imaging study reporting on 14 patients. Patient characteristics included (baseline/1 year): average age: 62/63±12, male: 64%, 64% and 50% history of hypertension and diabetes, dialysis modality: 57% hemodialysis and 43% PD, education above grade 12: 64%, and the average dialysis vintage was 41/54±60 months (Supplemental Table 1). This study was approved by Research Ethics Boards and conducted in accordance with the Declaration of Helsinki ethical standards.

Neurocognitive assessments were performed at both time points and included: Montreal Cognitive Assessment (dementia screening tool), the Trails Making Test (attention and executive function), and Creyos (electronic cognitive battery). Creyos was included to ensure capture of mild cognitive impairment, as dementia screening tools may underestimate cognitive impairment in dialysis populations.^4^ Full assessment battery descriptions are presented in Supplemental Table 2. All imaging and neurocognitive assessments were completed before dialysis.

It was determined that patients receiving dialysis experienced progressive WMI after a year of dialysis (*P* value: 0.00167, Figure 1A). When differences of FA values were analyzed in the 20 specific WM areas, all areas were significantly different between baseline and 1-year scans, indicating widespread WMI (Figure 1B and Supplemental Figure 1). Of note, when percent change of FA values was considered between the two scans, progressive WMI was apparent in all individuals receiving hemodialysis, along with one patient on PD (determined by Figure 1A and represented at least 9% change in FA value, Supplemental Table 3). The remaining patients receiving PD did not experience progressive WMI. When analyzed by WMI versus no WMI, there were significant difference in 18/20 Johns Hopkins University WM atlas areas (Figure 1B). This indicated that the group characterized as having progressive WMI acquired widespread WMI, whereas the non-WMI group only experience injury in Corticospinal Tract-R and Hippocampus-L. There was no significant difference in cognitive assessment scores between the two time points (baseline and 1 year) and groups (WMI and no WMI; Supplemental Table 4). Although there was no difference in scores between scans, overall reasoning domain and a task measuring reasoning and short-term memory (Creyos, Odd One Out Task) were associated with WMI after a Pearson correlation matrix was compiled (Supplemental Figure 2).

**Figure 1:**
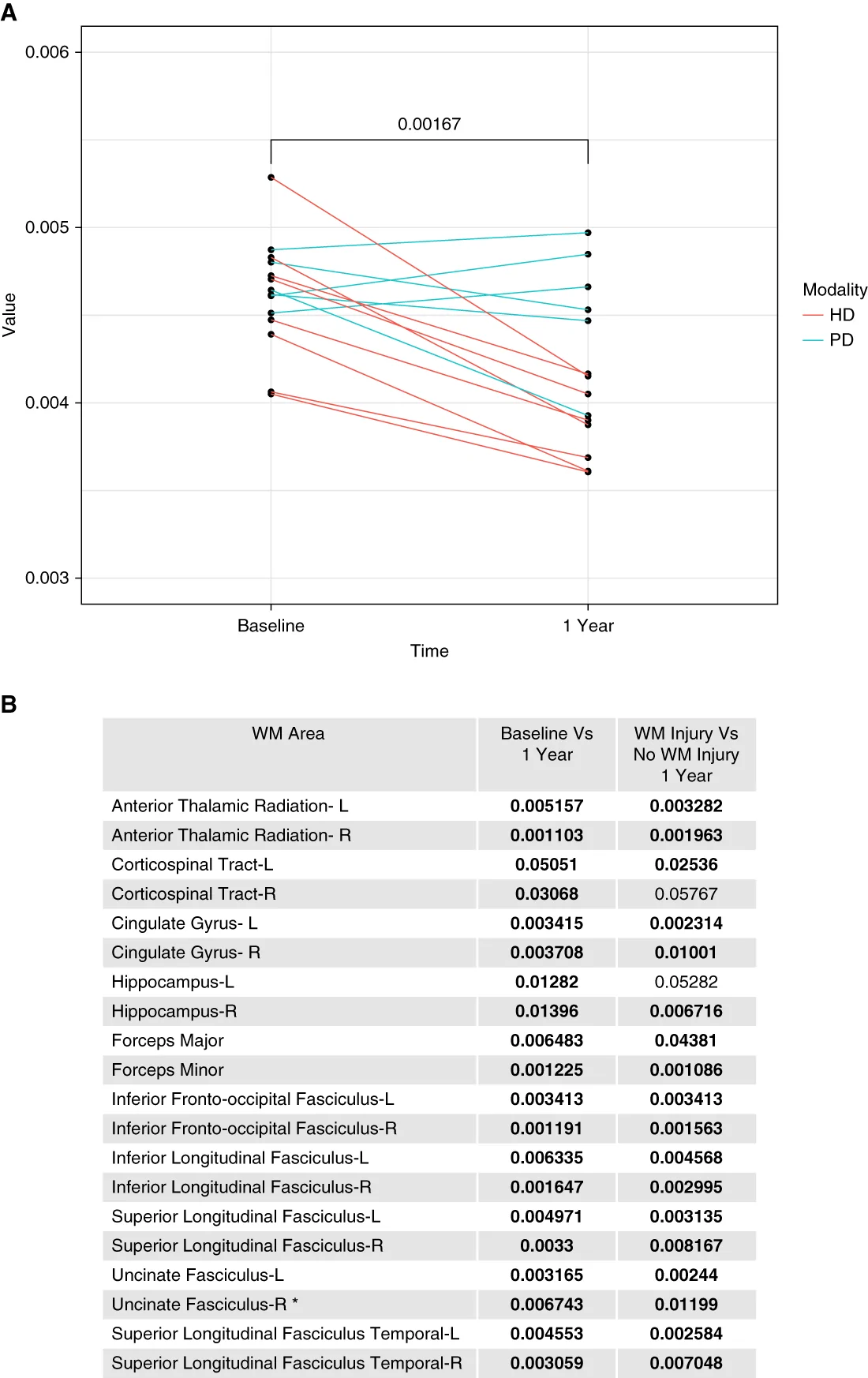
**Patients receiving hemodialysis experienced more progressive WMI then those on PD after 1 year of dialysis.** (A) DTI analysis for FA. FA analysis indicates significant WMI between a baseline dialysis scan and a follow-up scan 1 year later for 14 patients (eight hemodialysis, six PD) *P* value = 0.00167. Patients receiving hemodialysis experienced more progressive WMI then those on PD after 1 year of dialysis. (B) JHU WM atlas analysis. WM areas investigated *via t* test to determine significant FA values (bolded, *P* value < 0.05) between baseline and 1-year scans at a group level, and between those with WMI/progression and those without WMI/progression. All WM areas showed significant difference at a group level between baseline and 1 year. All areas except two (corticospinal tract-R, hippocampus-L) were significantly different between WMI/progression and no WMI/progression groups. *Uncinate fasciculus-R was the only WM area that was analyzed with Wilcoxon rank test after normality and variance were assessed, all other areas were analyzed *via t* tests. DTI, diffusion tensor imaging; FA, fractional anisotropy; HD, hemodialysis; JHU, Johns Hopkins University; PD, peritoneal dialysis; WM, white matter; WMI, white matter injury.

In conclusion, these findings indicate that whole-brain WMI is occurring over a year of dialysis, regardless of dialysis modality. Specifically, however, patients receiving hemodialysis exhibit progressive WMI compared with those who receive PD. This is congruent with previous findings that patients receiving PD did not experience intradialytic WMI,^5^ unlike patients receiving hemodialysis.^1^ Fewer hemodynamic fluctuations are thought to be the responsible for why patients on PD^6^ occur less circulatory stress. Although no differences were seen in cognitive assessment scores, associations between WMI and reasoning domain were determined. The lack of significant cognitive impairment between scans is likely due to the small cohort, and these patients were receiving dialysis prior to their baseline scan and may have already acquired cognitive impairment. Furthermore, these results highlight the implications of hemodialysis causing systemic harm, which has been previously reported.^7^ Advancements in the protection of patients receiving hemodialysis should be at the forefront of investigation in order to mitigate widespread cerebral damage that ultimately affects cognitive function and influences quality of life and health care burden.

## Supplementary Material

## Data Availability

Original data generated for the study will be made available upon reasonable request to the corresponding author. Data Type: Image Data. Reason for Restricted Access: Patient level data for cognition is available in supplemental material. Imaging data files are large and can be requested.

## Declarative Statements

This study includes clinical experimentation and received Institutional Review Board or Ethics Committee approval. All patients provided written informed consent. This study includes clinical experimentation and complies with the Declaration of Helsinki.

## Supplemental Material

Supplemental Table 1. Patient characteristics.

Supplemental Table 2. Neurocognitive battery test descriptions.

Supplemental Table 3. Percent change in FA skeletonized value between baseline and 1-year scans.

Supplemental Table 4. Cognitive outcomes.

Supplemental Figure 1. FA significant areas between baseline and 1-year DTI scans.

Supplemental Figure 2. Correlation between WMI and cognitive battery.
