# Supplementary material for: Progressive White Matter Injury Dominates in Patients Receiving Hemodialysis over Peritoneal Dialysis after 1 Year of Dialysis
Source: Kidney360. 2026 Mar 19;7(8):1880–3. doi: 10.34067/KID.0000001158 (PMC13567881; doi:10.34067/KID.0000001158)
Supplement: Supplementary file 1 [file kidney360-7-1880-s001.pdf]

ASN American Society of Nephrology
JASN Journal of the American Society of Nephrology
KIDNEY360 Accessing Our World From Every Angle
CJASN Clinical Journal of the American Society of Nephrology

ASN Journal Disclosure Form

As per ASN journal policy, I have disclosed any financial relationships or commitments I have held in the past 36 months as included below. I have listed my Current Employer below to indicate there is a relationship requiring disclosure. If no relationship exists, my Current Employer is not listed.

C. McIntyre reports the following:
Employer: University of Western Ontario; Consultancy: Vantive, NIPRO,, Sequana Medical,; Ownership Interest: uVOL ltd; Research Funding: Baxter, Sequana; Honoraria: Vantive, NIPRO; and Patents or Royalties: uVOL ttd.

I understand that the information above will be published within the journal article, if accepted, and that failure to comply and/or to accurately and completely report the potential financial conflicts of interest could lead to the following: 1) Prior to publication, article rejection, or 2) Post-publication, sanctions ranging from, but not limited to, issuing a correction, reporting the inaccurate information to the authors' institution, banning authors from submitting work to ASN journals for varying lengths of time, and/or retraction of the published work.

Name: Christopher W. McIntyre
Manuscript ID: k360-2025-00134r1
Manuscript Title: progressive white matter injury
Date of Completion: January 23, 2026
Disclosure Updated Date: October 3, 2025

ASN American Society of Nephrology JASN Journal of the American Society of Nephrology KIDNEY360 Accessing Our World From Every Angle CJASN Clinical Journal of the American Society of Nephrology

ASN Journal Disclosure Form

As per ASN journal policy, I have disclosed any financial relationships or commitments I have held in the past 36 months as included below. I have listed my Current Employer below to indicate there is a relationship requiring disclosure. If no relationship exists, my Current Employer is not listed.

J. Vanderlinden has nothing to disclose.

I understand that the information above will be published within the journal article, if accepted, and that failure to comply and/or to accurately and completely report the potential financial conflicts of interest could lead to the following: 1) Prior to publication, article rejection, or 2) Post-publication, sanctions ranging from, but not limited to, issuing a correction, reporting the inaccurate information to the authors' institution, banning authors from submitting work to ASN journals for varying lengths of time, and/or retraction of the published work.

Name: Jessica Vanderlinden
Manuscript ID: K360-2025-001334R1
Manuscript Title: Progressive White Matter Injury Dominates in Patients Receiving Hemodialysis Over Peritoneal Dialysis After One Year of Dialysis
Date of Completion: January 2, 2026
Disclosure Updated Date: September 18, 2025
