# Supplementary material for: Progressive White Matter Injury Dominates in Patients Receiving Hemodialysis over Peritoneal Dialysis after 1 Year of Dialysis
Source: Kidney360. 2026 Mar 19;7(8):1880–3. doi: 10.34067/KID.0000001158 (PMC13567881; doi:10.34067/KID.0000001158)
Supplement: Supplementary file 2 [file kidney360-7-1880-s002.pdf]

**Supplemental Material**

Supplemental Table 1: Patient Characteristics

| **Patient Characteristics** | **Baseline (N=14)** | **1-Year (N=14)** |
|---|---|---|
| **Age (Mean, Range)** | 62.2 (39-81) | 63.3 (40-82) |
| **Gender (N, %)** | | |
| Male<br>Female | 9 (64%)<br>5 (36%) | 9 (64%)<br>5 (36%) |
| **Education (N, %)** | | |
| Greater Than Grade 12<br>Less Than Grade 12 | 9 (64%)<br>5 (36%) | 9 (64%)<br>5 (36%) |
| **Cause of Chronic Kidney Disease (N)** | | |
| Diabetes<br>Hypertension<br>Other [a] | 6*<br>5*,**<br>8** | 6*<br>5*,**<br>8** |
| **Co-Morbidities (N)*** ** | | |
| Diabetes<br>Hypertension<br>Cardiovascular [b]<br>Other [c] | 6<br>10<br>6<br>28 | 6<br>10<br>6<br>28 |
| **Treatment Modality (N, %)** | | |
| **Hemodialysis**<br>Central Line<br>Fistula | 8 (57%)<br>6 (75%)<br>2 (25%) | 8 (57%)<br>6 (75%)<br>2 (25%) |
| **Peritoneal Dialysis**<br>Continuous Ambulatory Peritoneal Dialysis<br>Automated Peritoneal Dialysis | 6 (43%)<br>2 (33%)<br>4 (67%) | 6 (43%)<br>2 (33%)<br>4 (67%) |
| **Dialysis Vintage (Months) (Mean, Range)** | | |
| Group | 41 (7-243) | 54 (20-246) |

Supplemental Table 1: Patient characteristics and demographics

* Four patients had their cause of kidney disease as multifactorial, resorting in an extra count in the categories of diabetes/hypertension.

** One patient had their cause of kidney disease as multifactorial, resorting in an extra count in the categories of hypertension/Other.

*** All patients had more then one comorbidity leading to extra counts in each category. Note that if a patient’s cause of kidney disease included diabetes or hypertension, it was also counted in the comorbidities section.

a- Cause of Kidney Disease Other Category: Glomerular Nephritis (N=2), Vascular Disease (N=1), Lithium Toxicity (N=1), Norwalk Virus (N=1), Interstitial Nephritis (N=1), Focal Segmental Glomerulosclerosis (N=1), Acute Kidney Injury (N=1).

b- Comorbidities Cardiovascular Category: Heart Failure (N=2), Coronary Artery Disease (N=2), Heart Valve Replacement (N=1), Cardio-Renal Syndrome (N=1).

c- Comorbidities Other Category: Dyslipidemia (N=5), Sleep Apnea (N=4), Gastroesophageal Reflux Disease- GERD (N=4), Cancer (N=4, Breast, Skin, Renal, and Prostate all N=1), Neuropathy (N=3), Gout (N=2), Benign prostatic hyperplasia (BPH) (N=1), Pancreatitis (N=1), Bipolar (N=1), Retinopathy (N=1), Acute Tubular Necrosis (N=1), Glomerulonephritis (N=1).

**Supplemental Table 2:** Neurocognitive Battery Test Descriptions

| Neurocognitive Test | Neurocognitive Task/Test Description |
|---|---|
| MoCA | A dementia screening tool that measures domains of visuospatial/executive, naming, attention, memory, language, abstraction, orientation, and delayed memory. |
| Trails Making Test A | Patient is asked to connect the numbers 1 through 25 as quickly as possible, measuring visuomotor tracking, scanning, and attention. |
| Trails Making Test B | Patient is asked to alternate number and letters (ex.1-A-2-B-3-C), measuring mental shifting and executive function. |
| Double Trouble | A variation of the Stroop Task. Participants must pick which word describes the colour of the word depicted. |
| Grammatical Reasoning | Based on Alan Baddeley’s three-minute grammatical reasoning test. A picture of 2 shapes appears along with a sentence describing the relationship between the 2 shapes. Participants must determine if the sentence correctly describes the picture depicted. |
| Odd One Out Max | Based on a sub-set of problems from the Cattell Culture Fair Intelligence Test. Nine patterns are presented on the screen, and can differ in either shape, number or colour. The participant’s job is to determine the rules of the pattern to determine which shape does not fit the rule/pattern. |
| Monkey Ladder | Based off a task from non-human primate literature. Boxes appear in random locations on the screen and then light up with a number sequence. Once the last numbered box is displayed the number vanishes and the participant is asked to click the boxes in the correct order. Difficulty increases if the previous trial was correct and decreases if the previous trial was incorrect. |
| Token Search | Based on task measuring search strategy behaviours. Boxes are displayed around the screen. One box contains a token. Once located the participant is asked to find it again the remaining boxes. This continues until the token is located in each box displayed. Of note the token will never reappear in a box in which it has already appeared in. |
| Spatial Span | Based off Corsi Block Tapping Task. 16 squares are displayed in a 4 by 4 matrix. A random sequence of squares will light up in a predetermined order. Once the sequence is complete the participant is asked to repeat the sequence in the exact order. |
| Spatial Planning | Based on the Tower or London Task. Numbered circles appear on a tree-like structure out of order. The participant’s goal is to reposition the numbered circles in order on the tree in as few moves as possible. |
| Rotations | Two grids of colored squares in various patterns are depicted side by side, with one of the squares being rotated 90 degrees. Participants must determine if the images are the same or not. |
| Feature Match | Based on a Feature Search Task. Two grids made up of shapes appear on the screen and the participant must decide if the grids are identical or not. |

| | |
|---|---|
| Polygons | Based on the Interlocking Pentagons Task. A pair of overlapping polygons are displayed. Another polygon is depicted, and the participant must determine if it is identical to one of the overlapping polygons. |
| Digi Span | Based off a component of the WAIS-R intelligent test. A number sequence appears in a designated box. After the sequence is done participants must repeat the sequence in order on a dedicated number pad. |
| Paired Association | Based off a paradigm task. Boxes are displayed around the screen that contain different pictures. The pictures inside each box are shown and then hidden. Once all objects are shown and then hidden, one of the symbols is displayed in the middle of the screen and the participant must indicate where that picture is in the boxes. |
| Reasoning Domain | A combination score of all tasks using each task scores weighted reasoning value that is standardized by sex and age. |
| Verbal Domain | A combination score of all tasks using each task scores weighted verbal value that is standardized by sex and age. |
| Short-Term Memory Domain | A combination score of all tasks using each task scores weighted short-term memory value that is standardized by sex and age. |

**Supplemental Table 2:** Neurocognitive battery test descriptions. CBS descriptions were abstracted from: Sciences B. Cambridge Brain Sciences Tests Overview[1].

**Abbreviations:** Trails Making Test (TMT), The Wechsler Adult Intelligence Scale (WAIS-R).

**Supplemental Table 3: Percent Change In FA Skeletonized Value Between Baseline and 1-Year Scans**

| PT | 1 | 2 | 3 | 4 | 5 | 6 | 7 | 8 | 9 | 10 | 11 | 12 | 13 | 14 |
|---|---|---|---|---|---|---|---|---|---|---|---|---|---|---|
| MODALITY | HD | PD | PD | PD | HD | HD | PD | HD | HD | PD | HD | PD | HD | HD |
| RESULT | Injury | No Injury | No Injury | No Injury | Injury | Injury | No Injury | Injury | Injury | No Injury | Injury | Injury | Injury | Injury |
| FA | -9.26 | 3.32 | 5.26 | -5.60 | -11.80 | -19.73 | -3.18 | -12.72 | -11.01 | 1.90 | -13.87 | -15.43 | -21.48 | -17.78 |
| ANTERIOR THALAMIC RADIATION-L | -10.26 | 4.04 | 8.85 | -4.72 | -11.25 | -19.60 | -3.06 | -14.15 | -12.11 | 0.45 | -15.24 | -1.39 | -19.84 | -17.07 |
| ANTERIOR THALAMIC RADIATION-R | -13.36 | 0.10 | 3.10 | -7.66 | -14.33 | -20.30 | -3.62 | -14.29 | -12.26 | 1.00 | -15.55 | -0.86 | -22.92 | -19.44 |
| CORTICOSPINAL TRACT-L | -0.03 | 5.55 | 8.01 | -4.42 | -5.59 | -12.53 | -1.09 | -4.69 | -2.31 | -0.60 | -7.64 | -1.38 | -12.53 | -8.20 |
| CORTICOSPINAL TRACT-R | -0.37 | 5.40 | 4.75 | -6.71 | -4.19 | -11.30 | -2.64 | -7.28 | -2.09 | 3.55 | -7.07 | -1.83 | -13.37 | -12.17 |
| CINGULATE GYRUS- L | -11.25 | 4.10 | 5.88 | -5.60 | -14.33 | -23.99 | -3.66 | -14.78 | -15.41 | 0.43 | -15.80 | -0.82 | -24.11 | -21.29 |
| CINGULATE GYRUS- R | -10.30 | 3.60 | 2.56 | -6.19 | -13.13 | -19.97 | -3.07 | -13.92 | -13.64 | 4.17 | -11.24 | -0.94 | -22.45 | -18.11 |
| HIPPOCAMPUS-L | 1.63 | 1.63 | 10.12 | -2.77 | -13.57 | -20.52 | -5.23 | -10.04 | -7.93 | 0.97 | -24.03 | -7.26 | -17.90 | -7.05 |
| HIPPOCAMPUS-R | -2.56 | 5.17 | 7.41 | -7.83 | -11.09 | -19.28 | -7.48 | -14.56 | -8.00 | 8.91 | -9.59 | -7.22 | -25.51 | -18.26 |
| FORCEPS MAJOR | -5.56 | 3.07 | 3.38 | -6.45 | -8.88 | -13.95 | -3.81 | -10.07 | -9.25 | 0.68 | -13.66 | -2.13 | -30.05 | -15.55 |
| FORCEPS MINOR | -18.99 | 0.25 | 4.47 | -5.54 | -18.45 | -27.31 | -3.15 | -18.81 | -19.25 | -0.23 | -18.97 | -0.29 | -23.02 | -23.91 |
| INFERIOR FRONTO-OCCIPITAL FASCICULUS-L | -11.39 | 3.76 | 7.38 | -4.13 | -15.04 | -22.71 | -1.44 | -15.92 | -14.47 | -0.38 | -18.26 | -1.19 | -20.53 | -18.40 |
| INFERIOR FRONTO-OCCIPITAL FASCICULUS-R | -15.49 | 0.42 | 3.08 | -7.07 | -16.74 | -22.73 | -5.30 | -15.91 | -14.94 | 3.69 | -17.65 | -0.74 | -22.57 | -23.53 |
| INFERIOR LONGITUDINAL FASCICULUS-L | -6.95 | 4.40 | 5.61 | -3.59 | -11.30 | -18.95 | 1.22 | -12.37 | -10.19 | 0.26 | -15.81 | -2.56 | -20.42 | -15.79 |
| INFERIOR LONGITUDINAL FASCICULUS-R | -13.95 | 2.71 | 4.05 | -8.00 | -12.64 | -21.57 | -5.74 | -15.63 | -12.34 | 3.14 | -16.49 | -0.81 | -21.00 | -21.14 |
| SUPERIOR LONGITUDINAL FASCICULUS-L | -8.64 | 3.93 | 6.54 | -4.42 | -12.02 | -20.50 | -1.59 | -10.88 | -12.19 | 0.98 | -16.37 | -2.15 | -20.35 | -17.30 |
| SUPERIOR LONGITUDINAL FASCICULUS-R | -12.76 | 1.03 | 3.29 | -7.16 | -11.77 | -21.31 | -4.01 | -15.57 | -10.77 | 5.43 | -14.64 | 0.99 | -20.94 | -22.15 |
| UNCINATE FASCICULUS-L | -15.85 | 2.94 | 10.32 | -4.82 | -20.19 | -27.90 | -4.77 | -18.41 | -17.13 | 0.57 | -20.91 | -0.83 | -19.54 | -23.05 |
| UNCINATE FASCICULUS-R | -22.50 | -2.02 | 0.72 | -4.57 | -23.79 | -28.11 | -2.30 | -21.16 | -21.90 | 1.56 | -23.89 | -1.78 | -19.54 | -26.23 |
| SUPERIOR LONGITUDINAL FASCICULUS TEMPORAL-L | -8.96 | 4.02 | 6.35 | -4.98 | -13.52 | -21.71 | -0.99 | -12.22 | -11.45 | 1.10 | -16.54 | -2.13 | -19.20 | -16.86 |

| | | | | | | | | | | | | | | |
|---|---|---|---|---|---|---|---|---|---|---|---|---|---|---|
| **SUPERIOR LONGITUDINAL FASCICULUS TEMPORAL-R** | -14.04 | 0.86 | 3.27 | -7.17 | -12.57 | -22.34 | -3.47 | -17.08 | -11.13 | 5.28 | -14.93 | 1.61 | -18.98 | -22.30 |

**Supplemental Table 3:** Percent change in mean FA skeletonized data between baseline and 1-year scans. Injury result was determined by figure A in the manuscript indicating more than and 9% change in FA value between scans. Negative values indicate lower FA values which represents white matter injury.

**Supplemental Table 4: Cognitive Outcomes**

| *Test* | *Baseline Vs 1-Year P-Value* | *Test Type* | *WM Injury Vs No WM Injury 1-Year P-Value* | *Test Type* |
|---|---|---|---|---|
| *TMTA* | 0.4416 | Wilcoxon Rank Test | 0.6216 | T-Test |
| *TMTB* | 0.979 | Wilcoxon Rank Test | 0.9435 | T-Test |
| *MOCA* | 0.4148 | T-Test | 0.7679 | T-Test |
| *REASONING* | 0.2365 | T-Test | 0.4063 | T-Test |
| *VERBAL* | 0.617 | T-Test | 0.8977 | T-Test |
| *STM* | 0.7435 | T-Test | 0.7041 | T-Test |
| *PA* | 0.0893 | T-Test | 0.1379 | T-Test |
| *DS* | 0.6293 | T-Test | 0.969 | T-Test |
| *POLY* | 0.2874 | T-Test | 0.3264 | T-Test |
| *FM* | 0.5369 | T-Test | 0.5975 | T-Test |
| *ROT* | 0.0903 | T-Test | 0.4402 | T-Test |
| *SS* | 0.2755 | Wilcoxon Rank Test | 0.8013 | T-Test |
| *SP* | 0.7159 | T-Test | 0.7652 | T-Test |
| *TS* | 0.5362 | Wilcoxon Rank Test | 0.6285 | T-Test |
| *ML* | 0.8643 | T-Test | 0.6093 | T-Test |
| *GR* | 0.2698 | T-Test | 0.05407 | T-Test |
| *DT* | 0.9393 | Wilcoxon Rank Test | 1 | Wilcoxon Rank Test |
| *OOO* | 0.3907 | T-Test | 0.3402 | T-Test |

**Supplemental Table 4:** Statistical outcomes between baseline and 1-year, and grouping baseline and 1-year scores for those with progressive white matter injury and without white matter injury with regards to cognitive assessment scores. Test type was determined by assessing normality via Shapiro-Wilk Test and variance. No significant differences were found.

**Abbreviations:** WM: White Matter, TMTA/B: Trails Making Test Form A/B, MoCA: Montreal Cognitive Assessment, STM- Short-Term Memory, PA: Paired Association, DS: Digit Span, POLY: Polygons, FM: Feature Match, ROT: Rotations, SS: Spatial Span, SP: Spatial Planning, TS: Token Search, ML: Monkey Ladder, GR: Grammatical Reasoning, DT: Double Trouble, OOO: Odd One Out,

**Supplemental Figure 1: FA Significant Areas Between Baseline and 1-Year DTI Scans**

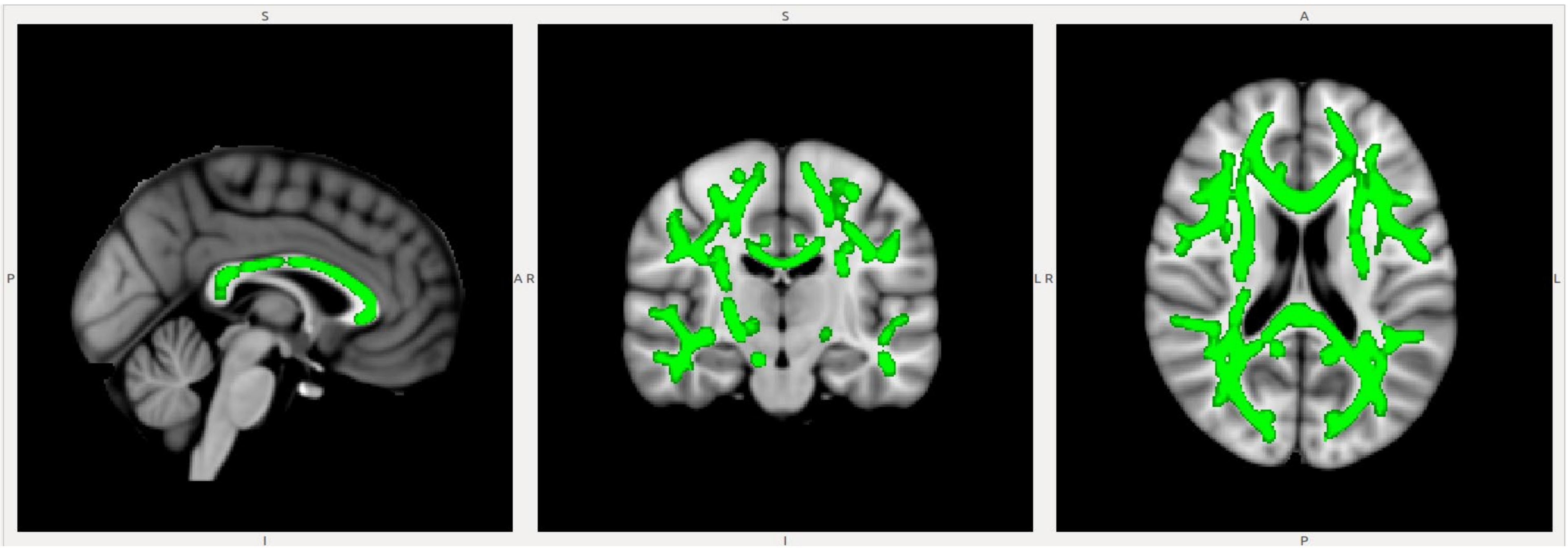


**Supplemental Figure 1**: Average significant differences in FA between baseline and 1-year DTI scans for all patients. This represents white matter differences between scans.

Note: DTI Analysis Plan: WMI was determined by diffusion tensor imaging acquired using a 3T Siemens MRI scanner using a 16-channel (12 head, 4 neck) phased array radiofrequency coil. Raw DTI images were optimized using Pierrick Coupe denoising package and corrected for head movement via Gibb's ringing, eddy current, and bias field corrections. Intensity inhomogeneities corrections were performed using FMRIB's Software Library (FSL), MRtrix3, and Advanced Normalization Tools. Corrected images received tensor fitting using non-linear least squares in Explore DTI, which resulted in fractional anisotropy (FA) scalar maps. FA maps were normalized to a MNI $T_1$ 1mm template, with Tract-Based Spatial Statistics analysis

being conducted in FSL to determine if there were differences between the two timepoints. Significant FA differences between timepoints were obtained via higher level/non-time series design for general linear models for paired groups. Further analysis, gathered white matter (WM) mean skeletonized FA values using the Johns Hopkins University (JHU) ICBM-DTI-81 WM labels atlas.

**Supplemental Figure 2: Correlation Between White Matter Injury and Cognitive Battery**

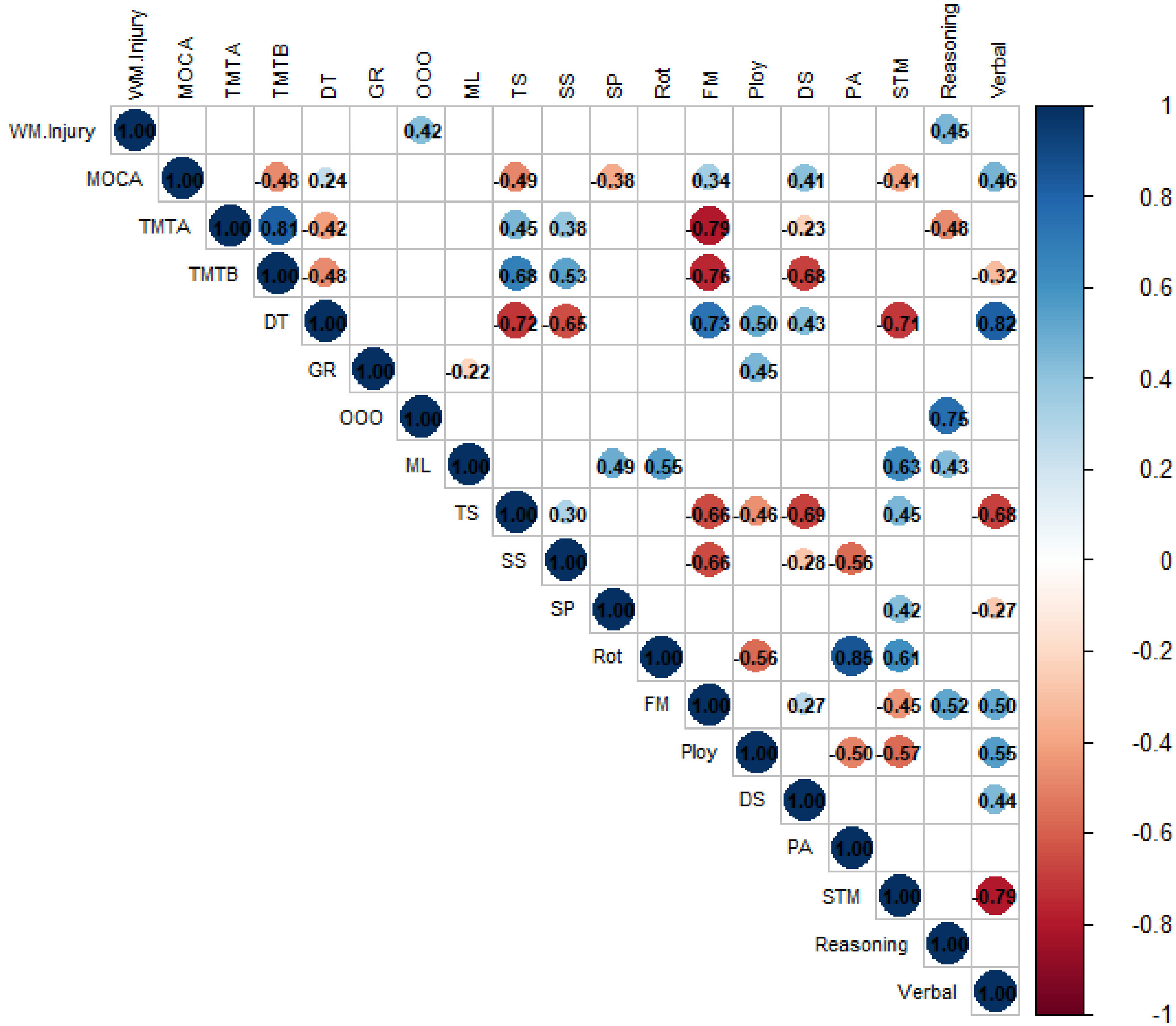

**Supplemental Figure 2:** Pearsons's correlation matrix between white matter injury and cognitive battery at 1-year. White matter injury was positively associated with odd one out and reasoning domain of the electronic Creyos assessment. Note: blue circles indicate positive correlations and red represents negative correlations. Strength of the correlation is determined by intensity of the colour.

**Abbreviations:** WM: White Matter, MoCA: Montreal Cognitive Assessment, TMTA/B: Trails Making Test Form A/B, DT: Double Trouble, GR: Grammatical Reasoning, OOO: Odd One Out, ML: Monkey Ladder, TS: Token Search, SS: Spatial Span, SP: Spatial Planning, ROT: Rotations, FM: Feature Match, POLY: Polygons, DS: Digit Span, PA: Paired Association, STM- Short-Term Memory.
